# The systemic environment: at the interface of aging and adult neurogenesis

**DOI:** 10.1007/s00441-017-2715-8

**Published:** 2017-11-09

**Authors:** Lucas K. Smith, Charles W. White, Saul A. Villeda

**Affiliations:** 1 Department of Anatomy, University of California, San Francisco, San Francisco, CA 94143 USA; 2 The Eli and Edythe Broad Center of Regeneration Medicine and Stem Cell Research, University of California San Francisco, San Francisco, CA 94143 USA; 3 Biomedical Sciences Graduate Program, University of California San Francisco, San Francisco, CA 94143 USA; 4 Developmental and Stem Cell Biology Graduate Program, University of California San Francisco, San Francisco, CA, 94143 USA

**Keywords:** Aging, Rejuvenation, Adult neurogenesis, Blood, Immune cells

## Abstract

Aging results in impaired neurogenesis in the two neurogenic niches of the adult mammalian brain, the dentate gyrus of the hippocampus and the subventricular zone of the lateral ventricle. While significant work has characterized intrinsic cellular changes that contribute to this decline, it is increasingly apparent that the systemic environment also represents a critical driver of brain aging. Indeed, emerging studies utilizing the model of heterochronic parabiosis have revealed that immune-related molecular and cellular changes in the aging systemic environment negatively regulate adult neurogenesis. Interestingly, these studies have also demonstrated that age-related decline in neurogenesis can be ameliorated by exposure to the young systemic environment. While this burgeoning field of research is increasingly garnering interest, as yet, the precise mechanisms driving either the pro-aging effects of aged blood or the rejuvenating effects of young blood remain to be thoroughly defined. Here, we review how age-related changes in blood, blood-borne factors, and peripheral immune cells contribute to the age-related decline in adult neurogenesis in the mammalian brain, and posit both direct neural stem cell and indirect neurogenic niche-mediated mechanisms.

## Introduction

Aging is a global process, resulting in disrupted homeostatic function and a loss of neurogenic capacity in the mammalian brain. Systemic interventions have recently been shown to regulate these effects in a variety of tissues, including the brain. Here, we will review the growing body of work that posits the systemic environment as a key regulator of the decline in neurogenesis observed in the aging mammalian brain, and will explore potential mechanisms by which the pro-aging and rejuvenating effects of the systemic milieu are mediated.

Traditionally, the adult mammalian brain was thought to be devoid of neural stem cell (NSC) activity, reliant largely on gene expression changes and structural reorganization for plasticity. In 1965, however, Altman and Das (1965) first observed evidence of newborn neurons in the adult rat hippocampus and, later, subventricular zone (SVZ). While controversial at the time, much work has since revealed that neurogenesis occurs throughout life in all vertebrate classes (Barker et al. 2011), including fish (Zupanc and Sirbulescu 2013), birds (Nottebohm 2004), reptiles (Chapouton et al. 2007) and in mammals ranging from rodents to humans (Amrein et al. 2011; Bond et al. 2015). In the adult mammalian hippocampus, neurogenesis occurs in a distinct region known as the dentate gyrus (DG). Specifically, NSCs involved in this process reside in the subgranular zone (SGZ), a thin, highly vascularized, layer of cells between the hilus and the densely populated granule cell layer. NSCs first enter a proliferative neuroblast state, replicating for a short time before beginning to migrate into the granule cell layer where they extend neuronal processes, taking on an immature neuronal identity after 2 weeks. Subsequently, mature differentiated neurons functionally integrate into the hippocampal circuit projecting to the CA3 after 4 weeks. In addition to the hippocampus, adult neurogenesis is observed in the SVZ of the mammalian brain. The NSC population of the SVZ resides along the walls of the lateral ventricles, in close proximity to the vasculature and cerebrospinal fluid (CSF). During adult neurogenesis, these cells adopt a proliferative neuroblast state and enter the rostral migratory stream, a path extending from the SVZ to the olfactory bulb. Upon reaching the olfactory bulb, these cells adopt either a granule or periglomerular cell fate, and migrate into the granule cell layer or glomerular layer of the olfactory bulb, respectively. In the years since its discovery, the physiological significance of adult neurogenesis has been further elucidated, with implications for cognitive function. Indeed, in the hippocampus, ablation of neurogenesis in mice has been shown to impair spatial learning and memory, pattern separation, and mood regulation (Zhang et al. 2008; Clelland et al. 2009; Deng et al. 2009; Christian et al. 2014; Tsai et al. 2015), while loss of SVZ neurogenesis leads to impaired odor discrimination (Valley et al. 2009; Sahay et al. 2011).

Neurogenesis has been shown to decrease in the hippocampus and SVZ of the adult mammalian brain as a function of age (Bizon et al. 2004; Enwere 2004; Amrein et al. 2011). Importantly, aging results in a structural remodeling of the DG and SVZ neurogenic niches, characterized by astrogliosis, microgliosis, and vascular remodeling, all of which may contribute to the decline in neurogenesis. Recently, much work has been carried out to understand how combinations of age-related changes in both the niche and intrinsic cellular processes play a role in this loss of neurogenesis. In the hippocampus, a significant increase in the ratio of quiescent to active NSCs accompanies age, and suggests that a loss of NSC activity rather than number plays a role in cognitive decline (Lugert et al. 2010). At the neurogenic niche level, several age-related changes have been linked to changes in neurogenesis, including loss of fibroblast growth factor-2 and vascular endothelial growth factor signaling (Shetty et al. 2005), decreased Wnt signaling (Seib et al. 2013), and increases in bone morphogenic protein (BMP) signaling (Yousef et al. 2015a). In the olfactory bulb, age-dependent loss of function has been shown to result from a marked reduction in the capacity of cells in the niche to undergo neurogenesis in response to epidermal growth factor signaling (Enwere 2004). Furthermore, an age-dependent increase in p16^INK4a^, an intrinsic factor commonly associated with senescence, within the progenitor population has also been shown to specifically impair neurogenesis within the SVZ (Molofsky et al. 2006). At a functional level, the role of the age-related decline in neurogenesis in mediating the age-related impairments in cognitive function remains controversial (Drapeau et al. 2003; Merrill et al. 2003; Seib et al. 2013). Notwithstanding, interventions that boost adult neurogenesis are consistently accompanied by cognitive enhancements in aged mice (Kempermann et al. 2002; van Praag et al. 2005; Kodali et al. 2015), raising the prospect that identifying regulators of age-related decline in neurogenic capacity could reveal potential targets for cognitive improvement at old age.

## Systemic environment: regulator of adult neurogenesis with age

While cell-intrinsic and niche-related factors are important in the age-related decline in neurogenesis, it is increasingly appreciated that the systemic environment also plays a role in regulating stem cell function during aging. Studies using the model of heterochronic parabiosis, in which the circulatory systems of a young and aged mouse are surgically joined, have allowed for a deepened understanding of systemic regulation and coordination of aging in multiple tissues (reviewed in Castellano et al. 2015). To date, researchers have revealed that exposure to old blood inhibits regeneration in muscle (Conboy et al. 2005; Brack et al. 2007; Rebo et al. 2016) and liver (Conboy et al. 2005), while young blood exerts rejuvenating effects on bone repair (Baht et al. 2015), β-cell proliferation (Salpeter et al. 2013), cardiac hypertrophy (Loffredo et al. 2013), muscle (Conboy et al. 2005; Brack et al. 2007; Sinha et al. 2014), and liver (Conboy et al. 2005). Importantly, both the pro-aging effects of old blood and rejuvenating effects of young blood extend to the CNS (Villeda et al. 2011, 2014; Ruckh et al. 2012; Baruch et al. 2014; Katsimpardi et al. 2014; Rebo et al. 2016; Castellano et al. 2017).

Given the close proximity of adult NSCs to the vasculature of the brain (Fuentealba et al. 2012), it is perhaps unsurprising that adult NSCs are highly sensitive to exposure to old and young blood. Indeed, exposure to old blood in the model of parabiosis, leads to decreased proliferation in both the DG and SVZ (Villeda et al. 2011; Katsimpardi et al. 2014). Furthermore, young heterochronic parabionts exhibited a decrease in the numbers of neural progenitor cells (NPCs) in the DG and SVZ, accompanied by a decrease in differentiated mature neurons (Villeda et al. 2011; Katsimpardi et al. 2014). Conversely, young blood leads to increased NPC proliferation and neuronal differentiation and survival in both the DG and SVZ of aged heterochronic parabionts (Villeda et al. 2011; Katsimpardi et al. 2014). Interestingly, exposure to young blood appears to drive stable rejuvenating changes in aged NSCs, as SVZ NSCs isolated from old heterochronic parabionts exhibit increased proliferation and increased neurogenesis when cultured in vitro, compared to NSCs isolated from old isochronic controls (Katsimpardi et al. 2014). Together, these studies indicate that neurogenesis is regulated by blood in an age-dependent manner through effects on NSC function.

Recently, the anti-neurogenic effects of aged blood were further corroborated in an independent model of heterochronic blood exchange, in which the circulatory system of young and old mice are connected via a jugular venous catheter, and blood repeatedly exchanged over the course of 24 h (Rebo et al. 2016). Short-term exposure to old blood in this model led to a decrease in proliferating NPCs in the DG of young mice (Rebo et al. 2016). Interestingly, short-term exposure to young blood in this model did not recapitulate the rejuvenating effects observed in old heterochronic parabioints (Villeda et al. 2011; Katsimpardi et al. 2014), where young blood exposure is more long-term. These findings suggest that the rejuvenating effects of young blood on neurogenesis may only be apparent after prolonged exposure.

## Blood plasma: loss of pro-youthful and accumulation of pro-aging soluble factors

Given the exciting rejuvenating effects observed in the model of heterochronic parabiosis, there is much interest in identifying blood-borne soluble factors that might underlie the pro-youthful effects of young blood. While intravenous (I.V.) administration of young adult blood plasma recapitulates aspects of heterochronic parabiosis (Villeda et al. 2014), its effects on neurogenesis have not yet been investigated. Despite this, GDF11, a TGF-β superfamily member, has emerged as a potential pro-youthful factor that boosts SVZ neurogenesis in old mice (Katsimpardi et al. 2014). GDF-11 was originally shown to decline in the blood of aged mice, and to rejuvenate aging phenotypes in heart (Loffredo et al. 2013) and muscle (Sinha et al. 2014); however, its effects on muscle remain under discussion (Egerman et al. 2015; Hinken et al. 2016). More work is warranted to further identify systemic rejuvenating factors that can promote neurogenesis in the aged brain.

In addition to identifying pro-youthful factors in young blood that might boost neurogenesis, an emerging body of work has identified aged blood plasma, and the factors contained therein, as a potent driver of brain aging. Consistent with the effects observed in heterochronic parabiosis and catheter-based blood exchange studies, blood plasma isolated from aged mice exerts anti-neurogenic effects on hippocampal neurogenesis when administered by I.V. injection in young mice (Villeda et al. 2011). Interestingly, old serum inhibits NSC function, in vitro, leading to decreased self-renewal (Villeda et al. 2011) and NPC proliferation (Bickford et al. 2015) in rodent models. Together, these data indicate the existence of soluble pro-aging factors that inhibit neurogenesis.

Multianalyte profiling of blood plasma from young and old mice, as well as from young isochronic and heterochronic parabionts, has identified six factors (B2M, CCL11, CCL12, CCL19, CCL2, and Haptoglobin) that are elevated in young heterochronic parabionts, and whose blood concentrations inversely correlate with the age-related decline in neurogenesis (Villeda et al. 2011). Of these factors, B2M, a non-covalently bound subunit of MHC class 1, and the chemokines CCL11 and CCL2 have been shown to be elevated in the blood of aged healthy humans (Targowski et al. 2005; Shurin et al. 2007; Mátrai et al. 2009; Villeda et al. 2011; Smith et al. 2015; Valiathan et al. 2016). Independent of their canonical immune functions, B2M, CCL11, and CCL2 have all been implicated as potential negative regulators of adult hippocampal neurogenesis (Villeda et al. 2011; Lee et al. 2013; Smith et al. 2015). For example, while it is yet unclear if the systemic increase of CCL2 with age contributes to the decline in adult neurogenesis, in the context of recovery from irradiation, CCL2 knockout mice exhibited increased neurogenesis after cranial irradiation (Lee et al. 2013). To date, mimicking an aged systemic environment by increasing either B2M or CCL11 has been shown to impair hippocampal neurogenesis in young mice (Villeda et al. 2011; Smith et al. 2015). In particular, systemic administration of B2M resulted in a decrease in the number of NPCs, immature neurons, and newly formed mature neurons in the hippocampi of young mice (Smith et al. 2015). Similarly, young mice given systemic injections of CCL11 had decreased adult neurogenesis compared to vehicle-treated controls (Villeda et al. 2011). Both B2M and CCL11 also inhibit neurogenesis when locally administered by stereotaxic injection into the DG of the hippocampus, and administration of B2M or CCL11 in vitro also inhibits NPC function (Villeda et al. 2011; Smith et al. 2015). Together, these studies suggest that these factors are able to directly regulate NSC function. Supporting this possibility, CCL11 readily crosses the blood–brain barrier (BBB) (Erickson et al. 2014). Additionally, while it is unknown whether B2M crosses the BBB, exposure to old blood through parabiosis or catheter-based blood exchange leads to elevated levels of B2M in the hippocampi of young mice (Smith et al. 2015; Rebo et al. 2016).

In addition to the factors identified through parabiosis, a number of other potential pro-aging factors have been identified, such as TGF-β, IL-6, and TNF-α. Indeed, TGF-β levels in blood are elevated in aged mice and systemic attenuation of TGF-β by pharmacological inhibition of its receptor, Alk5, resulted in increased hippocampal neurogenesis in aged mice that had also received a muscle injury (Yousef et al. 2015b). While administration of TGF-β1 to cultured NPCs has been shown to inhibit proliferation (Buckwalter et al. 2006; Yousef et al. 2015b) suggesting a direct regulatory effect of TGF-β signaling on neurogenesis, inhibition of TGF-β signaling in vivo was accompanied by a decrease in hippocampal B2M expression (Yousef et al. 2015b), suggesting that TGF-β may influence neurogenesis through multiple mechanisms*.* Additionally, aging is accompanied by increased IL-6 and TNF-α in the blood of humans (Bruunsgaard 2006; Valiathan et al. 2016). As both these factors have been shown to inhibit murine NPC function in vitro, (Ben-Hur et al. 2003; Monje et al. 2003), it is possible that they further contribute to the age-related decline in neurogenesis. Whether preventing the age-related accumulation of these pro-aging factors can boost neurogenesis in the aged brain remains to be determined.

**Fig. 1:**
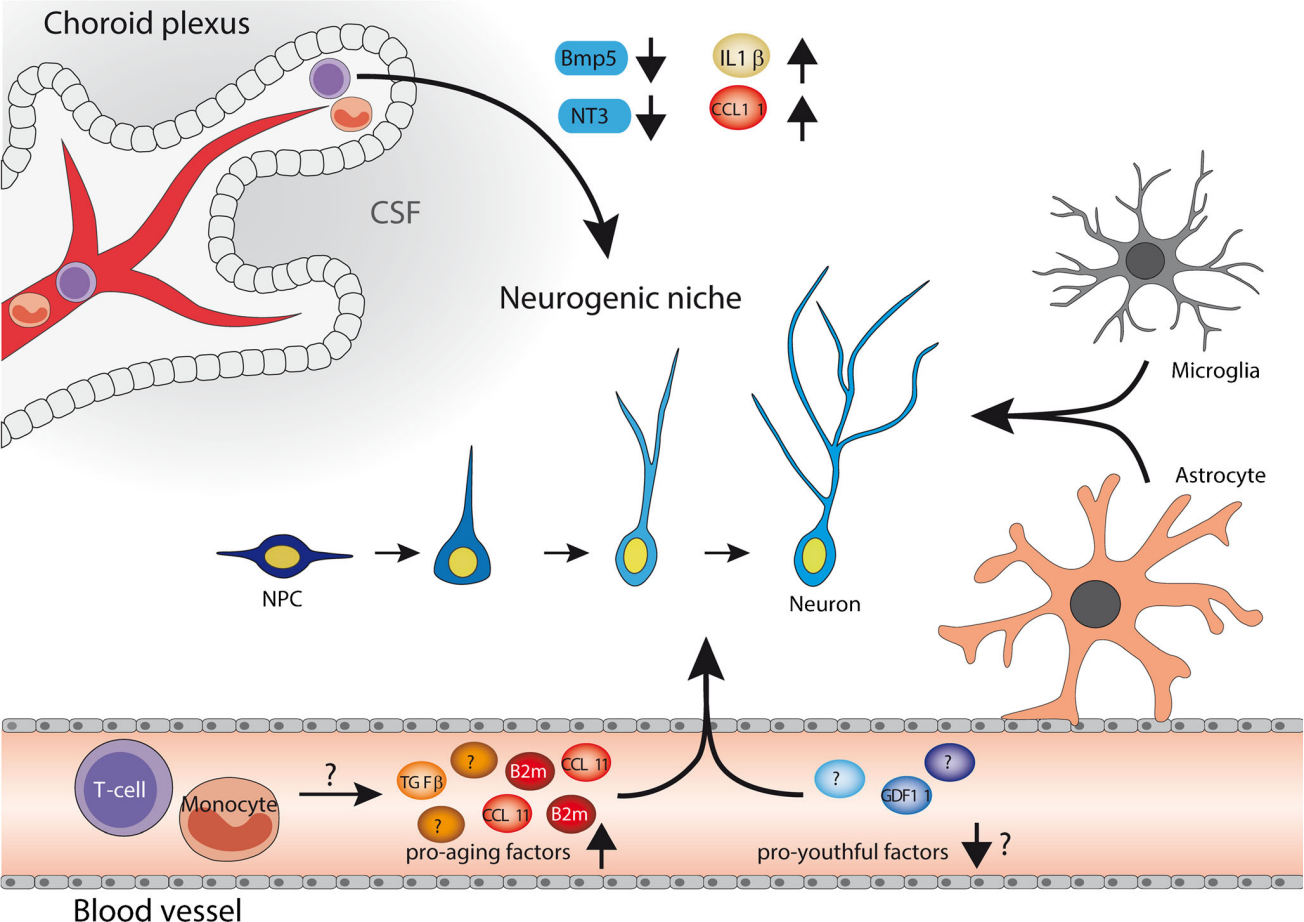
Potential mechanisms by which the aging systemic environment regulates the age-related decline in neurogenesis in the adult brain. Schematic illustration highlighting possible mechanisms by which age-related changes in blood may regulate neurogenesis in the aged mammalian brain. Age-related changes in immune cells (T cells and monocytes) and soluble factors in aging blood may regulate neurogenesis directly by modulating neural stem/progenitor cell function, or indirectly by altering signaling in the local neurogenic niche and choroid plexus.* Arrows* denote potential pathways by which blood aging affect neurogenesis. Mechanisms yet to be determined are denoted by a question mark (*?*).* NPC* neural progenitor cell;* CSF* cerebral spinal fluid;* B2m* β2-microglobulin;* TGFβ * transforming growth factor;* CCL11* C-C motif chemokine 11;* NT3* neurotrophin 3;* BMP5* bone morphogenetic protein 5;* IL-1β* interleukin 1β

Based on these studies, a model has emerged in which mammalian aging drives a decline in pro-youthful factors and concomitant accumulation of detrimental pro-aging immune factors in blood that direct the age-related decline in neurogenesis (Fig. 1) (Bouchard and Villeda 2015). While in vitro studies suggest that pro-aging factors can directly affect NPC function, more research is warranted to determine the mechanisms by which pro-aging factors exert their anti-neurogenic affects. Furthermore, the underlying cellular sources responsible for the accumulation of pro-aging factors in old blood remain unknown. However, given their immune origin, it is possible that accumulation of pro-aging factors reflects a pro-inflammatory remodeling of the peripheral immune system.

## Immune cells: loss of neurotrophic and gain of anti-neurogenic function

Aging drives many functional and structural changes in the hematopoietic system, resulting in impaired immune function (immunosenescence), increased rates of anemia, and increased incidence of myeloid malignancies (Wahlestedt et al. 2015). Specifically, aging results in decreased production of naïve T cells, and peripheral expansion of senescent memory and effector T cells (Dorshkind et al. 2009) that may promote an inflammatory systemic milieu as a result of their elevated cytokine production (Effros 2007; Huang et al. 2008; Montecino-Rodriguez et al. 2013). Similarly, there is reduced production of B cells, and an accumulation of memory cells with increased production of autoreactive antibodies (Dorshkind et al. 2009). Accompanying these changes to the adaptive immune system is the progressive dysregulation of the innate immune system, which includes the functional decline of neutrophils, natural killer cells, monocytes/macrophages, and dendritic cells (Shaw et al. 2010)*.* Together, these changes are poised to contribute to the accumulation of pro-aging factors in old blood, and thereby inhibit neurogenesis (Fig. 1). Indeed, increased transcription of two potential pro-aging factors, B2M and IL-6, have been reported in aged human peripheral blood mononuclear cells (PBMCs) (Snyder-Mackler et al. 2014).

In addition to the possibility that pro-inflammatory changes to the immune system leads to increased levels of pro-aging factors, it is possible that the aging hematopoietic system also loses neurotrophic properties with age (Fig. 1). This idea has gained traction given the emerging data demonstrating a pro-neurogenic role of peripheral blood immune cells in young adult mice. For example, mice lacking either Ly6C(hi) monocytes or T cells have impaired neurogenesis, and adoptive transfers of CD4+, but not CD8+, T cells, into T cell-deficient mice enhances neurogenesis (Ziv et al. 2006; Wolf et al. 2009; Möhle et al. 2016). Given that monocyte populations and T cells have both been shown to exhibit functional decline during age (Dorshkind et al. 2009; Shaw et al. 2010), it is possible that cellular aging of the immune system results in a loss of neurotrophic function during age (Fig. 1). Thus, it has been hypothesized that rejuvenating the immune system could counter brain aging and promote adult neurogenesis (Ron-Harel and Schwartz 2009). Supporting this hypothesis, it has been demonstrated that a single intravenous injection of human umbilical cord blood mononuclear cells (hUCBMC) can increase proliferation and neuronal differentiation in the DG of the hippocampi in aged but not young rats (Bachstetter et al. 2008). Interestingly, in similar experiments, administration of adult PBMCs had no effect on neurogenesis, indicating that the young age of the mononuclear cells is critical for its pro-neurogenic effects (Bachstetter et al. 2008). Furthermore, consistent with previous work suggesting a neurotrophic function of CD4+ T cells (Ziv et al. 2006; Wolf et al. 2009), the rejuvenating effects of hUCBC transplantation were later recapitulated by a single dose of human umbilical cord blood (hUCB)-derived CD4+ T cells, and not by administration of other types of mononuclear cells (Shahaduzzaman et al. 2013). At the cellular level, cultured rat NSCs grown in hUCB-derived T cell-conditioned media exhibited increased proliferation and viability compared with conditioned media from other hUCBMCs (Shahaduzzaman et al. 2013), suggesting that the effect could be mediated by secreted factors from the young T cells. While the mechanism by which hUCB cells promote neurogenesis remains to be elucidated, given that peripheral blood T cells and monocytes are rarely found in the brain parenchyma of healthy mice, it is likely that the effects of the immune system on neurogenesis in the aged brain are mediated through indirect mechanisms affecting the blood–brain interface.

## Choroid plexus: reflections of blood aging at the blood-brain interface

The choroid plexus (CP) is a highly vascular tissue located in the ventricles of the mammalian brain that produces CSF and acts as an interface between the periphery and the brain. Secreted factors from the CP bathe the SVZ neurogenic niche and are thought to influence neurogenesis in development and adulthood (Lehtinen et al. 2013). Moreover, SVZ NSCs contact CSF directly, via apical processes (Fuentealba et al. 2012)*,* highlighting their vulnerability to changes in the CSF. Aging drives an inflammatory transcriptional profile in the CP (Baruch et al. 2014; Silva-Vargas et al. 2016), and has been associated with an upregulation of type I interferon (IFN-I) dependent gene expression and decreased IFN-II expression (Baruch et al. 2014). Furthermore, aging results in an altered secretome by the CP, which may result in decreased neurotrophic capacity. Recently, it has been shown that aging CP-conditioned media contains decreased levels of BMP5 and IGF-1 that normally promote neurogenesis in the SVZ (Silva-Vargas et al. 2016). The aged CP also exhibits elevated expression of IL-6 and CCL11 (Baruch et al. 2013), both of which have been shown to inhibit neurogenesis (Monje et al. 2003; Villeda et al. 2011). Additionally, CCL11 and B2M levels are also elevated in CSF from aged humans (Villeda et al. 2011; Smith et al. 2015). Together, these studies support a model in which age-related changes in the CP lead to changes in the composition of soluble factors in the CSF, which contributes to the age-related decline in adult neurogenesis.

Recent evidence now suggests a direct functional role for CP aging and age-related decline in neurogenesis in the brain. For example, conditioned media from aged CP explants inhibits adult SVZ neurogenesis compared to young CP-conditioned media when infused into the lateral ventricles of young mice in vivo, or administered on young SVZ NSCs in vitro (Silva-Vargas et al. 2016). Conversely, in aged mice, young CP-conditioned media rejuvenates adult SVZ neurogenesis in vivo, and enhances self-renewal of aged SVZ NPCs in vitro (Silva-Vargas et al. 2016). Interestingly, activated NSCs and not later progenitor populations appear to be highly sensitive to age-related changes in CP-conditioned media, as heterochronic infusions of CP-conditioned media affected the numbers of proliferating SVZ NSCs in vivo, but not numbers of proliferating progenitors (Silva-Vargas et al. 2016). Furthermore, in vitro, purified activated NSCs were highly sensitive to the effects of heterochronic CP-conditioned media on colony formation while transit-amplifying cells and quiescent NSCs were not (Silva-Vargas et al. 2016). In addition to affecting SVZ neurogenesis, CP aging also influences hippocampal neurogenesis. Inhibition of the age-related IFN-I expression profile, through administration of an IFN-I receptor neutralizing antibody to the CSF of aged mice, increased the number of proliferating cells and immature neurons in the DG of aged mice (Baruch et al. 2014).

Given its highly vascular nature, it is likely that the CP may be sensitive to changes in soluble pro-aging factors in blood. Additionally, CD4+ T cells have recently been observed in the CP of healthy young and aged mice (Baruch et al. 2013), suggesting a potential sensitivity to cellular changes in the aged immune system. Supporting this idea, in the model of heterochronic parabiosis, blood age was found to modulate IFN-II expression profiles in the CP of old and young mice (Baruch et al. 2014). Interestingly, however, age-related differences in IFN-I profiles remained unchanged in parabiosis (Baruch et al. 2014), indicating that blood aging may contribute to some but not all age-related changes observed in the CP (Baruch et al. 2014). Of note, mice deficient for IFN-II signaling due to the absence of an IFN-y receptor exhibited decreased adult hippocampal neurogenesis (Baruch et al. 2014), suggesting that the transcriptional changes in IFN-II signaling at the CP induced by young or aged blood may be of functional consequence in mediating their effects on neurogenesis. Furthermore, young mice exposed to aged blood exhibited elevated CP-expression of the pro-aging factor, CCL11, compared to isochronic controls (Baruch et al. 2014), positing additional mechanisms by which blood aging may act through the CP to regulate adult neurogenesis. Together, these studies position the CP as a potential link between the systemic milieu and neurogenesis, implicating the CP as a regulator of the age-related decline in neurogenesis (Fig. 1).

## Neurogenic niche: mediator of the aging systemic environment?

The neurogenic niche is complex, comprised of many different cell types that influence NSC function (reviewed by Aimone et al. 2014). Vasculature, microglia, and astrocytes have all been shown support adult neurogenesis under basal conditions; however, this homeostatic relationship can be disrupted, particularly in the context of inflammation (Leiter et al. 2016). Thus, it has been postulated that age-related changes to the neurogenic niche—which include astrogliosis, microgliosis and vasculature deterioration—may contribute to the age-related decline in adult neurogenesis (Leiter et al. 2016). Given the inflammatory remodeling that occurs in blood during aging, it is possible that the age-related changes in the neurogenic niche at least partially reflect blood aging, thereby providing an additional mechanism by which blood aging could regulates neurogenesis. In line with this, age-related changes in the systemic milieu have also been shown to regulate cellular components of the neurogenic niche. In particular, in parabioisis studies, exposure to young blood increased blood vessel volume and branching accompanied by increased neurogenesis in the SVZ of aged mice (Katsimpardi et al. 2014). Furthermore, aged mice systemically administered GDF11 exhibited increased blood vessel volume, along with increased SVZ neurogenesis (Katsimpardi et al. 2014).

In addition to modulating vasculature, age-related changes in the systemic milieu may also regulate neuroinflammation, and thereby affect neurogenesis by impairing the homeostatic capacity of microglia and astrocytes. While aging is associated with microgliosis, adoptive transfer of hUCB mononuclear cells or T cells decreased the number of activated microglia in the hippocampus of old rats, suggesting that young immune cells may dampen neuroinflammation in the aged neurogenic niche (Bachstetter et al. 2008; Shahaduzzaman et al. 2013). Conversely, given the immune origin of the pro-aging factors identified in old blood to date, it seems likely that old blood may also promote neuroinflammation and, consequently, inhibit neurogenesis. This would be consistent with heterochronic parabiosis and blood exchange studies demonstrating elevated B2M expression in the hippocampi of young mice exposed to old blood, which could reflect an increase in neuroinflammation (Smith et al. 2015; Rebo et al. 2016). Correspondingly, attenuation of TGF-B in old mice also dampens B2M expression in the hippocampi of aged mice (Yousef et al. 2015b). These data suggest that neurogenic niche may mediate the effects of heterochronic parabiosis on neurogenesis (Fig. 1); however, the full effects of aged blood on the neurogenic niche have not been fully investigated. Thus, a more thorough evaluation of the effects of blood on the neurogenic niche is warranted to determine whether and how blood aging broadly affects cellular hallmarks of brain aging.

## Conclusion

A growing body of evidence has demonstrated that the systemic environment is a key mediator of age-related decline in neurogenesis. Models of heterochronic blood exchange have revealed that the age-related decline in adult neurogenesis in both the DG and SVZ can be either ameliorated or promoted by exposure to young or aged blood, respectively (Villeda et al. 2011; Katsimpardi et al. 2014). We propose a model in which blood aging results in both a loss-of-neurotrophic-function and a concomitant gain-of-detrimental-function that negatively regulate NSC function with age (Fig. 1). At the molecular level, old blood plasma—and in particular soluble immune factors elevated therein, such as B2M and CCL11—inhibit adult neurogenesis (Villeda et al. 2011; Smith et al. 2015). At the cellular level, increasing evidence indicates that peripheral blood immune cells regulate neurogenesis in adult mice (Ziv et al. 2006; Wolf et al. 2009; Möhle et al. 2016), which may be disrupted by age-related immunosenescence. Furthermore, the CP, which serves as an interface between the brain and blood, has now been proposed as a potential mediator of these immune-related effects. Indeed, exposure to young and old blood modulates transcriptional hallmarks of CP aging (Baruch et al. 2014) that may in turn affect the CP secretome, shown to modulate neurogenesis in an age-dependent manner. At the neurogenic niche level, aging results in altered vasculature, microgliosis and astrogliosis, all of which can in turn regulate neurogenesis (Leiter et al. 2016). While exposure to young blood has been shown to increase vasculature in the aged mouse SVZ (Katsimpardi et al. 2014), the extent to which the aging systemic environment alters the neurogenic niche is not fully understood. Ultimately, while the systemic environment has now been identified at the interface of aging and adult neurogenesis, much work remains to fully elucidate the intricate and multifaceted mechanisms by which the systemic environment promotes loss of neurogenic capacity in the aged mammalian brain. Additionally, an emerging field of aging research is now looking to models of extended lifespan and delayed aging to explore the potential for rejuvenation. It will be of interest to examine the link between the aging systemic environment and neurogenesis in models in which aging process is protracted, such as the naked mole rat (Penz et al. 2015), in order to gain insights into protective mechanisms that delay the aging process.
